# Involvement of Nitrogen on Flavonoids, Glutathione, Anthocyanin, Ascorbic Acid and Antioxidant Activities of Malaysian Medicinal Plant *Labisia pumila* Blume (Kacip Fatimah)

**DOI:** 10.3390/ijms13010393

**Published:** 2011-12-29

**Authors:** Mohd Hafiz Ibrahim, Hawa Z. E. Jaafar, Asmah Rahmat, Zaharah Abdul Rahman

**Affiliations:** 1 Department of Crop Science, Faculty of Agriculture, University Putra Malaysia, Serdang, Selangor 43400, Malaysia; E-Mail: mhafizphd@yahoo.com; 2 Department of Nutrition & Dietetics, Faculty of Medicine & Health Sciences, University Putra Malaysia, Serdang, Selangor 43400, Malaysia; E-Mail: asmah@medic.upm.edu.my; 3 Department of Land Management, Faculty of Agriculture, University Putra Malaysia, Serdang, Selangor 43400, Malaysia; E-Mail: zaharah@agri.upm.edu.my

**Keywords:** *Labisia pumila* Blume, nitrogen fertilization, plant secondary metabolites, gluthatione, DPPH radical scavenging, ferric reducing antioxidant power

## Abstract

A split plot 3 by 4 experiment was designed to characterize the relationship between production of gluthatione (GSH), oxidized gluthatione (GSSG), total flavonoid, anthocyanin, ascorbic acid and antioxidant activities (FRAP and DPPH) in three varieties of *Labisia pumila* Blume, namely the varieties *alata*, *pumila* and *lanceolata*, under four levels of nitrogen fertilization (0, 90, 180 and 270 kg N/ha) for 15 weeks. The treatment effects were solely contributed by nitrogen application; there was neither varietal nor interaction effects observed. As the nitrogen levels decreased from 270 to 0 kg N/ha, the production of GSH and GSSG, anthocyanin, total flavonoid and ascorbic acid increased steadily. At the highest nitrogen treatment level, *L. pumila* exhibited significantly lower antioxidant activities (DPPH and FRAP) than those exposed to limited nitrogen growing conditions. Significant positive correlation was obtained between antioxidant activities (DPPH and FRAP), total flavonoid, GSH, GSSG, anthocyanin and ascorbic acid suggesting that an increase in the antioxidative activities in *L. pumila* under low nitrogen fertilization could be attributed to higher contents of these compounds. From this observation, it could be concluded that in order to avoid negative effects on the quality of *L. pumila*, it is advisable to avoid excessive application of nitrogen fertilizer when cultivating the herb for its medicinal use.

## 1. Introduction

Epidemiology studies increasingly recommend that eating of a diet rich in plant foods acts as a defense against cardiovascular disease and certain forms of cancer [1]. Although a variety of plant components including proteins, amino acids, vitamins, and fiber may lead to overall health benefits, recent research has focused on the role of secondary plant metabolites, particularly flavonoid compounds, in disease prevention [2]. These plant carbon based secondary metabolites (CBSM) can vary widely in their structure and general classification, but they all share the common feature of containing at least one aromatic ring and one or more hydroxyl groups [3].

Flavonoid compounds in plants are naturally occurring antioxidants, and their radical scavenging capabilities are thought to play an important function in preventing many chronic illnesses [3,4]. They have been shown to inhibit metastasis and tumorigenesis [5,6], and many are known to have anti-inflammatory, antibacterial and antifungal capabilities [7]. These effects are mainly attributed to their antioxidant activity. Antioxidants are substances that delay or inhibit oxidative damage when present in small quantities compared to an oxidizable substrate [8]. Antioxidants affect the process of lipid peroxidation due to the differences in their form of action. Hence, antioxidants can help in disease prevention by effectively neutralizing the free radicals or inhibiting damage created by them [9]. Plant antioxidants are believed to play a role in protection against a variety of diseases and to delay ageing processes. The health promoting effect of antioxidants from plants could be due to their protective effects by counteracting reactive oxygen species (ROS) [10]. There are several compounds which contribute to the antioxidative properties; these include polyphenols [11], vitamin C [12], anthocyanins [13] and flavonoids [14].

Research is uncovering the fact that the availability of plant nutrients can be important factors in determining secondary metabolism and antioxidant within plants [15,16]. Nitrogen is one of the most important growth factors in controlling yield and quality of plants. Moreover, nitrogen modulates the biosynthesis of secondary metabolites (e.g., flavonoid compounds, glucosinate, carotenoid, *etc*.) [17]. Nitrogen (N) supply has a negative effect on the biosynthesis of flavonoids and chlorogenic acid in plants. Bongue and Phillips [18] reported that nitrogen (N) deficit increased the level of total flavonoids by 14% in tomato. However, in grapefruit, the concentration of the flavonoids naringin and rutinoside decreased in the fruit with increased N supply [19]. Furthermore, Awad and de Jager [20] found that the total flavonoids and chlorogenic acid concentrations in apple skin decreased with increasing of N supply. While N is an essential nutrient element for crop growth and quality, little is known about the effect of N supply on the antioxidant activity of medicinal plants.

Among these medicinal plant species, *Labisia pumila* Blume (Myrsinacea family), or known locally as Kacip Fatimah in Malaysia, has been given particular attention. It is a popular herb that has been recognized to contain high flavonoids contents [21,22]. Both phenolic acids and flavonoids are believed to be responsible for the wide spectrum of pharmacological activities attributed to this herb [23]. The plant has been used as a medicinal treatment for dysentry, flatulance, dysmonorrhea and gonorrhoea [24]. Previous studies on *L. pumila* performed with different nitrogen fertilizations have shown that high nitrogen can reduce the production of secondary metabolites in this herb due to reduced phenyl alanine lyase (PAL) activity that was correlated with low C/N ratio, photosynthetic rates and total non structural carbohydrate (TNC) [25]. However, documentation of the phytochemical properties of *L. pumila* is still lacking, especially the antioxidative capacities of *L. pumila* to different nitrogen fertilization has not been reported. This information is important and will be useful in the cultivation as well as in the preparation of herbal formulations for health supplements. Therefore, a study was carried out to determine antioxidant activity, antioxidant scavenger (GSH, GSSG), total flavonoid, antocyanin and vitamin C of methanolic extracts from three varieties of *L. pumila*, namely *L. pumila* var. *alata*, *L. pumila* var. *pumila* and *L. pumila* var. *lanceolata* under different N fertilization. The relationships among the parameters of GSH, GSSG, antocyanin, vitamin C and antioxidant activities were also investigated.

## 2. Results and Discussion

### 2.1. Total Flavonoid Profiling

Nitrogen fertilization had a significant (*P* ≤ 0.01) impact on the production of total flavonoids (Table 1). There were no varietal and interaction effects observed. As more nitrogen was invested from 0 to 270 kg N/ha, the amount of total flavonoids produced decreased. This plant accumulated more secondary metabolites in the leaves, followed by the stem and then roots. In the leaves, as nitrogen fertilization decreased from 180 to 90 and 0 kg N/ha, the total flavonoid content was enhanced by 3, 13 and 32%, respectively, compared to 270 kg N/ha. The increase of total plant flavonoids and phenolics under limited N fertilization was also reported in previous studies by Felgines *et al.* [26] and Koricheva *et al.* [27]. Increase in carbon based secondary metabolites (CBSM) under low N fertilization was in agreement with the Carbon Nutrient Balance (CNB) theory by Bryant *et al.* [28], who predicted the increase in production of flavonoids under low N fertilization. The increase in flavonoids under low N fertilization might be attributed to increase in phenylalanine (phe) availability due to restriction of protein synthesis under N deficiency [20]. The enhanced phe would substantially enhance the production of flavonoids as phe is also a precursor for the formation of flavonoids [29]. Previous studies have shown that flavonoids content such as quercetin had anticancer activities and were able to inhibit cancer cell growth [30,31]. Quercetin was reported to have high scavenging activities and act as a treatment for hayfever, hives, sinusitis, asthma, and inflammation disorders [32,33]. Some studies also reported that quercetin plays an important role in the prevention of atherosclerosis [34]. The present result showed that quercetin content could be enhanced by low nitrogen fertilization to *L. pumila*.

### 2.2. Glutathione (GSH), Oxidised Glutathione (GSSG) and Ratio of GSH/GSSG Profiling

The GSH, GSSG and GSH/GSSG in *L. pumila* were influenced by Nitrogen levels (*P* ≤ 0.01; Table 2). The GSH, GSSG and GSH/GSSG ratio were found to have similar trend with flavonoids accumulation. The highest accumulation of GSH was found to be in the leaf at 0 kg N/ha that recorded 876.23 nmol gluthathione/g dry weight where the lowest was observed in the root at 270 kg N/ha that recorded 398.56 nmol glutathione/g dry weight. In GSSG, leaf—0 kg N/ha and root—270 kg N/ha recorded 200.76 and 54.67 nmol oxidised glutathione/g dry weight, respectively. For the GSH/GSSG ratio the root—270 kg N/ha recorded the highest GSH/GSSG (7.29) while leaf—180 kg N/ha depicted the lowest GSH/GSSG that only recorded 3.95. GSH is a tripeptide composed of cysteine, glutamic acid and glycine and is the most abundant nonprotein thiol in the cells. Its active group is the thiol (–SH) of cysteine. GSH is maintained in the reduced state. The GSH plays an imperative role in the stabilization of many enzymes. Additionally, as an antioxidant scavenger it serves as a substrate for Dehydroascorbate (DHAsA) reductase and is also directly reactive with free radicals including the hydroxyl radical to prevent the inactivation of enzymes by oxidation of an essential thiol group [35]. GSSG consists of two GSH molecules joined by their –SH group into a disulfide bridge and was found to be present in low quantities compared to GSH [36]. In the present study, we found that reduced N fertilization increased GSH and GSSG content. The high GSH and GSSG are necessary for several physiological functions. These include activation and inactivation of redox-dependent enzyme systems and regeneration of cellular antioxidant ascorbic acid under oxidative conditions [37,38]. Usually, the increase in GSH and GSSG in reduced N fertilization is associated with an increase in antioxidant properties [36]. In the current study, it was shown that GSH and GSSG have a strong positive relationship with total flavonoids, vitamin C and antocyanin content (Table 3). The result showed that the increase in antioxidative properties of *L. pumila* under low nitrogen fertilization might be due to an increase in production of total flavonoids, GSH and GSSG activity that can increase the antioxidant capacity of this plant under these condition [39,40].

### 2.3. Anthocyanin and Their Profiling

Anthocyanin content was found to be influenced by the application of nitrogen (*P* ≤ 0.01). The accumulation of anthocyanin was found to be highest in the leaves followed by the stems and lowest in roots. In the leaves, N fertilization at 0 kg N/ha (0.71 mg/g fresh weight), 90 kg N/ha (0.58 mg/g fresh weight) and 180 kg N/ha (0.38 mg/g fresh weight) had produced more antocyanin than at 270 kg N/ha, which registered a meager 0.19 mg/g fresh weight by the end of 15 weeks of experiment (Table 4). Also, in the roots there was only 0.11 mg/g fresh weight produced under 270 kg N/ha compared to 0.31 mg/g fresh weight at 180 kg N/ha, 0.47 mg/g fresh weight at 90 kg N/ha and 0.60 mg/g fresh weight at 0 kg N/ha. Similar findings were observed by Brunetto *et al.* [41] and Delgrado *et al.* [42] on grapevines. Usually anthocyanins accumulate under low N fertilization [43]. Bongue-Bartelsman and Phillips [18] demonstrated that N stress produces effects on expression of genes encoding enzymes associated with anthocyanin biosynthesis. Anthocyanins are the naturally occurring phenolic compounds responsible for the color of many flowers, fruits, and berries [43]. It is the most important group of water soluble pigments in plants and has beneficial health effects as antioxidant and antiinflammatory agents [44]. Anthocyanins are probably the largest group of phenolic compounds in the human diet, and their strong antioxidant activities suggest their importance in maintaining health. Anthocyanins are also important as antioxidants, which have roles in promoting good health and reducing the risk of chronic disease and also as anti-inflammatory agents. It was reported by Tamura and Yamagami [45] that anthocyanins possess some positive therapeutic effects, mainly associated with their antioxidant activities. In the current study, it was found that enhanced N fertilization can reduce the anthocyanin content, thus suggesting a decrease in the quality of *L. pumila* under excessive N fertilization.

### 2.4. Ascorbic Acid and Their Profiling

Ascorbic acid, also known as vitamin C, is one of the most abundant antioxidants in plant where the role of ascorbate is to protect plants against oxidative stress [46]. It is a powerful water soluble antioxidant and its established role is to prevent scurvy [47]. The profiling of ascorbic acid in *L. pumila* plants followed the same trend as the total flavonoids, gluthatione and anthocyanin content, where the availability of vitamin C was found to be higher in the leaves and lowest in roots (Table 4). The imposition of lower N levels has resulted in significantly higher ascorbic acid contents in the leaves, stems and roots of *L. pumila*. By the end of week 15 after start of treatments, the ascorbic acid contents in the leaves of plants receiving 0, 90 and 180 kg N/ha were 0.061, 0.049 and 0.029 mg/g l-ascorbic acid fresh weight, respectively, compared to only 0.017 mg/g l-ascorbic acid fresh weight achieved with 270 kg N/ha application. The same observation was found by Salomez and Hofman [48] and Staugaitis *et al.* [49] when they observed vitamin C content in lettuce and Chinese cabbage was substantially reduced with application of high N fertilizer. The increase in vitamin C content under low N application levels in *L. pumila* seedlings might possibly be attributed to low vegetative growth that decreased self-shading while increasing exposure to irradiance, hence, improving the production of vitamin C in the plant. According to Seung and Adel [50], vitamin C tends to accumulate more in plant parts that are exposed to sunlight; this justifies why there was increased production of vitamin C under low N fertilization.

### 2.5. Radical Scavenging Activity

Generally, DPPH antioxidant activity was highest in the leaves followed by stems and roots in all nitrogen application treatments. The treatment effects of DPPH were contributed by nitrogen levels (*P* ≤ 0.05; Table 5). At 350 μg/mL, the DPPH antioxidant activity recorded the highest value (61.32–51.21%) at 0 kg N/ha followed by the 90 kg N/ha (50.83–46.73%), 180 kg N/ha (46.43–40.21%), and the least in the 270 kg N/ha treatment (37.21–30.65%). However, DPPH radical scavenging abilities of the extracts of the plants were lower than those of butylated hydroxyl toluene (BHT; 61%) and α-tocopherol (76.31%) registered at 350 μg/mL. This study showed that *L. pumila* methanolic extract has a good free radical scavenging activity and, hence, it can be used as a radical scavenger, acting possibly as the primary antioxidant. This result also implies that high N supply could significantly reduce the DPPH radical scavenging activity of a medicinal plant. It is noteworthy that DPPH assay principally measures the activity of the water-soluble antioxidants [51].

The principle of this method is that in the presence of a molecule consisting of a stable free radical (DPPH), an antioxidant with the ability to donate a hydrogen atom will quench the stable free radical, a process which is associated with a change in the absorption and can be translated spectrophotometrically. To date, more than 8000 phenolic compounds are known in plants, of which almost two-thirds belong to the predominantly water soluble flavonoids antioxidant family. Results of the current work also suggest that high N supply was disadvantageous to *L. pumila* in the improvement of the antioxidant activity of water-soluble antioxidants. In our study, besides flavonoid compounds, other water-soluble antioxidants of the extracts such as ascorbic acid and anthocyanin could also exert an additive effect on DPPH radical scavenging activity. Many studies have shown that a combination of flavonoids compounds with anthocyanin and ascorbic acid produced a synergistic effect on DPPH radical scavenging activity [52].

### 2.6. Reducing Ability

The FRAP assay is very simple, fast and precise, and was recently developed to measure the total antioxidant power of biological fluids [53]. Total antioxidant power was assessed by the reduction of Fe^3+^ to Fe^2+^, which occurred rapidly with all reductants with half of the reaction reduction potentials above that of Fe^3+^/Fe^2+^. Therefore, the values express the corresponding concentration of electron-donating antioxidants. The Ferric reducing Antioxidant Potential (FRAP) was influenced by the nitrogen fertilization (*P* ≤ 0.01). The FRAP activity was found to be highest in 0 kg N/ha, followed by 90 kg N/ha, 180 kg N/ha and 270 kg N/ha (Table 6). In plant parts, the highest FRAP activity was observed in the leaves followed by the stems and the roots. The reducing ability of extracts from different parts of plants without any application of N (0 kg N/ha) was in the range of 890.32 to 810.21 μm of Fe(II) dry weight, while at 270 kg N/ha treatment the reducing ability of the extracts exhibited a range of 435.23 to 399.43 μm of Fe(II) dry weight (Table 5). The result indicates that fertilization with low N was able to possess high abilities to reduce Ferric Ions [24]. In the leaves, stems and roots, the antioxidant potential of *L. pumila* was estimated from their ability to reduce 2,4,6-tripyridyl-*s*-triazine (TPTZ)-Fe(III) complex to TPTZ-Fe(II). The FRAP values for the methanolics extracts of the leaves, stems and roots in all varieties were statistically and significantly lower than vitamin C and α-tocopherol, but higher than that of BHT.

The ferric reducing ability (FRAP assay) is widely used in the evaluation of the antioxidant component of dietary polyphenols [54]. The antioxidant activity is found to be linearly proportionate to the phenolics and flavonoids content [55]. Yen *et al.* [56] reported that the ferric reducing power of bioactive compounds was associated with antioxidant activity. Glenn *et al.* [57] have reported a strong positive relationship between total flavonoids compounds and antioxidant activity, which appears to be of similar trend shown by results of the current study where total flavonoids displayed significantly positive relationships with FRAP activity of *R*^2^ = 0.912 (*P* ≤ 0.05; Table 3). Furthermore, DPPH and FRAP had a significant positive relationship with GSH, GSSG, anthocyanin and ascorbic acid; this justifies that high DPPH and FRAP activity in *L. pumila* extract under low N levels might be due to high accumulation of GSH, GSSG, total flavonoids, anthocyanin and vitamin C in the plant [11–14].

## 3. Experimental

### 3.1. Experimental Location, Plant Materials and Treatments

This experiment was carried out in growth houses at Field 2, Faculty of Agriculture Glasshouse Complex, Universiti Putra Malaysia (longitude 101°44′ N and latitude 2°58′ S, 68 m above sea level) with a mean atmospheric pressure of 1.013 kPa. The experiment started from 10 July 2010 to 11 September 2010. About three-month old *L. pumila* seedlings of var. *alata*, *pumila* and *lanceolata* were left for a month to acclimatize in a nursery until ready for the experiments. The seedlings were planted in soilless medium containing coco-peat, burnt paddy husk and well composted chicken manure in 5:5:1 (v/v) ratio in 25-cm diameter polyethylene bags. Day and night temperatures in the greenhouse were maintained at 27–30 °C and 18–21 °C, respectively, and relative humidity from 50 to 60%. All the seedlings were irrigated using overhead mist irrigation given four times a day or when necessary. Each irrigation session lasted for 7 min. When the seedlings had reached 4 months of age, they were fertilized with four rates of nitrogen, *viz*. 0, 90, 180 and 270 kg N/ha, applied in the form of urea. The fertilization with nitrogen levels were split into three applications (Table 7). This factorial experiment was arranged in a split plot using a randomized complete block design with varieties being the main plot, and nitrogen levels as the sub-plot replicated three times. Each treatment consisted of 10 seedlings.

### 3.2. Total Flavonoids Quantification

The method of quantification for total flavonoids contents followed after Ibrahim and Hawa [58]. About 0.1 ground tissue samples was extracted with 80% ethanol (10 mL) on an orbital shaker for 120 min at 50 °C. The mixture was consequently filtered (Whatman™ No.1), and the filtrate was used for the determination of total flavonoids. For total flavonoids determination, a sample (1 mL) was mixed with NaNO_3_ (0.3 mL) in a test tube covered with aluminium foil, and left for 5 min. Then 10% AlCl_3_ (0.3 mL) was added followed by addition of 1 M NaOH (2 mL). Later, the absorbance was measured at 510 nm using a spectrophotometer with rutin as a standard (results expressed as mg g^−1^ quercetin dry sample).

### 3.3. Measurement of Glutathione (GSH) and Oxidized Glutathione (GSSG)

GSH and GSSG were assayed using the method described by Castillo and Greppin [59]. Total glutathione were determined by reacting 0.5 mL plant extracts with 50 mM KH_2_PO_4_/2.5 mM EDTA buffer (pH 7.5), 0.6 mM DTNB [5,5-dithio-bis-2-nitrobenzoic acid] in 100 mM Tris-HCl, pH 8.0, 1 unit of glutathione reductase (GR, from spinach, EC 1.6.4.2) and 0.5 mM NADPH. GSH was quantified from the reaction mixture by mixing 0.5 mL of plant extract with 60 mM KH_2_PO_4_/2.5 mM EDTA buffer (pH 7.5), 0.6 mM DTNB in 200 mM Tris-HCl, pH 8.0. The mixture was incubated at 30 °C for 15 min, and the reaction was followed as the rate of change in absorbance at 412 nm using a light spectrophotometer (UV-3101P, Labomed Inc, USA). GSSG was determined after removal of GSH from the plant extract.

### 3.4. Ascorbic Acid Content

The ascorbic acid content was measured using a modified method of Davis and Masten [60]. The fresh leaf samples (1 g) were extracted in 1% of phosphate-citrate buffer, pH 3.5 using a chilled pestle and mortar. The homogenate was filtered. The filtrate was added to the 1 mL of 1.7 mM 2,6-dichloroindophenol (2,6-DCPIP) in a 3 mL cuvette. The absorbance at 520 nm was read within 10 min of mixing the reagents. The extraction buffer was used as a blank. l-Ascorbic acid was used as a standard. Ascorbic acid was recorded as mg/g l-ascorbic acid fresh leaves.

### 3.5. Anthocyanin Content

Anthocyanin content was determined according to Bharti and Khurana [61]. Fresh leaves (1 g) were added in 10 mL acidic methanol (1% v/v HCl) and incubated overnight. Anthocyanin was partitioned from chlorophyll with 10 mL chloroform, followed by adding 9 mL of double deionised water. The test tubes containing the samples were shaken gently and the mixture allowed to settle. The absorbance was read at 505 nm. Petunidin was used as a standard. Anthocyanin content was recorded as mg/g petunidin fresh weight.

### 3.6. DPPH Radical Scavenging Assay

The DPPH free radical scavenging activity of each sample was determined according to the method described by Joyeux *et al.* [62]. A solution of 0.1 mM DPPH in methanol was prepared. The initial absorbance of the DPPH in methanol was measured at 515 nm. An aliquot (40 μL) of an extract was added to 3 mL of methanolic DPPH solution. The change in absorbance at 515 nm was measured after 30 min. The antiradical activity (AA) was determined using the following formula:

AA%=100-[(Abs:sample-Abs:empty sample)]×100)/Abs:control

The optic density of the samples, the control and the empty samples were measured in comparison with methanol. One synthetic antioxidant, BHT (butylhydroxytoluene) and α-tocopherol, were used as positive controls. The antioxidant capacity based on the DPPH free radical scavenging ability of the extract was expressed as μmol Trolox equivalent per gram of dried plant material.

### 3.7. Reducing Ability (FRAP Assay)

The ability to reduce ferric ions was measured using modifying methods of Ibrahim and Hawa [63]. An aliquot (200 μL) of the extract with appropriate dilution was added to 3 mL of FRAP reagent (10 parts of 300 mM sodium acetate buffer at pH 3.6, 1 part of 10 mM TPTZ solution and 1 part of 20 mM FeCl_3_ 6H_2_O solution) and the reaction mixture was incubated in a water bath at 37 °C. The increase in absorbance at 593 nm was measured after 30 min. The antioxidant capacity based on the ability to reduce ferric ions of the extract was expressed as expressed in μM Fe(II)/g dry mass and compared with those of standards for BHT, ascorbic acid, and α-tocopherol.

### 3.8. Statistical Analysis

Data were analyzed using analysis of variance using SAS version 17. Mean separation test between treatments was performed using Duncan multiple range test and standard error of differences between means was calculated with the assumption that data were normally distributed and equally replicated [64,65].

## 4. Conclusions

In conclusion, our results indicate that manipulation of N fertilization levels may be an effective method to increase the expression of secondary metabolites compounds in *L. pumila*. Higher total flavonoids, GSH, GSSG, anthocyanin content and ascorbic acid concentrations were observed in *L. pumila* when nutrient availability was limited by the non application of N fertilizer. Moreover, at the highest nitrogen level treatment, *L. pumila* exhibited significantly lower antioxidant activities (DPPH and FRAP) than those under limited N growing conditions. In order to avoid negative effects on the quality of *L. pumila*, it is recommended that no excess N application should be practiced when cultivating *L. pumila* for its medicinal use.

## Figures and Tables

**Table 1:** Accumulation and partitioning of total flavonoids (TF) in different plant parts of *Labisia pumila* Blume under different nitrogen levels.

Nitrogen levels	Plant parts	Total flavonoids (TF) (mg quercetin/g dry weight)
**0 kg N/ha**	Leaf	0.90 ± 0.02 ^a^
	Stem	0.77 ± 0.12 ^a^
	Root	0.55 ± 0.02 ^c^

**90 kg N/ha**	Leaf	0.77 ± 0.03 ^a^
	Stem	0.67 ± 0.04 ^b^
	Root	0.52 ± 0.06 ^c^

**180 kg N/ha**	Leaf	0.70 ± 0.07 ^b^
	Stem	0.63 ± 0.05 ^b^
	Root	0.50 ± 0.02 ^c^

**270 kg N/ha**	Leaf	0.68 ± 0.04 ^b^
	Stem	0.44 ± 0.08 ^d^
	Root	0.34 ± 0.01 ^e^

All analyses are mean ± standard error of mean (SEM). *N* = 18. Means not sharing a common letter are significantly different at *P* ≤ 0.05.

**Table 2:** Gluthathione (GSH), Oxidised Gluthatione (GSHO) and GSH/GSSG ratio in different part of *L. pumila* under different nitrogen levels.

Nitrogen levels	Plant parts	GSH (nmol/g dry wt)	GSSG (nmol/g dry weight)	GSH/GSSG
**0 kg N/ha**	Leaf	876.2 ± 11.2 ^a^	200.6 ± 9.8 ^a^	4.4 ± 0.6 ^d^
	Stem	766.5 ± 9.8 ^b^	145.2 ± 9.8 ^b^	5.3 ± 0.1 ^b^
	Root	435.2 ± 11.2 ^d^	87.7 ± 7.6 ^d^	5.0 ± 0.9 ^c^

**90 kg N/ha**	Leaf	778.2 ± 8.6 ^b^	187.5 ± 8.7 ^a^	4.2 ± 0.4 ^d^
	Stem	665.3 ± 13.5 ^c^	123.6 ± 9.5 ^c^	5.4 ± 0.7 ^b^
	Root	412.3 ± 6.8 ^d^	76.6 ± 6.7 ^e^	5.4 ± 0.6 ^b^

**180 kg N/ha**	Leaf	700.3 ± 7.8 ^b^	178.6 ± 7.3 ^a^	4.0 ± 0.1 ^e^
	Stem	612.3 ± 9.8 ^c^	121.5 ± 7.2 ^c^	5.0 ± 0.2 ^c^
	Root	399.6 ± 10.3 ^d^	65.7 ± 9.3 ^e^	6.0 ± 0.1 ^a^

**270 kg N/ha**	Leaf	689.5 ± 11.3 ^c^	156.7 ± 5.6 ^b^	4.4 ± 0.2 ^d^
	Stem	598.6 ± 9.8 ^c^	112.3 ± 6.8 ^d^	5.3 ± 0.3 ^b^
	Root	398.5 ± 13.3 ^d^	54.6 ± 7.3 ^d^	7.3 ± 0.2 ^a^

All analyses are mean ± standard error of mean (SEM). *N* = 18. Means not sharing a common letter are significantly different at *P* ≤ 0.05.

**Table 3:** Correlations among the measured parameters in the experiments.

Parameters	1	2	3	4	5	6	7

**1 Flavonoid**	1.000						
**2.GSSG**	0.823 **	1.000					
**3.GSH**	0.715	0.812 *	1.000				
**4.Antocyanin**	0.845 *	0.749 *	0.771 *	1.000			
**5. Vitamin C**	0.816 *	0.864 *	0.749 *	0.736 *	1.000		
**6. DPPH**	0.923 *	0.940 *	0.849 *	0.711 *	0.756 *	1.000	
**7. FRAP**	0.912 *	0.826 *	0.546	0.726 *	0.745 *	0.918 **	1.000

* and

** respectively significant at *P* ≤ 0.05 or *P* ≤ 0.01.

**Table 4:** Accumulation and partitioning of Antocyanin and Ascorbic Acid in different plant parts of *Labisia pumila* Blume under different Nitrogen levels.

Nitrogen levels	Plant parts	Anthocyanin (mg/g fresh weight)	Ascorbic acid (mg/g fresh weight)
**0 kg N/ha**	Leaf	0.71 ± 0.01 ^a^	0.061 ± 0.001 ^a^
	Stem	0.67 ± 0.02 ^a^	0.060 ± 0.021 ^a^
	Root	0.60 ± 0.03 ^a^	0.057 ± 0.012 ^a^

**90 kg N/ha**	Leaf	0.58 ± 0.12 ^b^	0.049 ± 0.021 ^b^
	Stem	0.51 ± 0.23 ^b^	0.045 ± 0.011 ^b^
	Root	0.47 ± 0.12 ^b^	0.041 ± 0.017 ^b^

**180 kg N/ha**	Leaf	0.38 ± 0.03 ^c^	0.029 ± 0.024 ^c^
	Stem	0.37 ± 0.03 ^c^	0.027 ± 0.009 ^c^
	Root	0.31 ± 0.02 ^c^	0.021 ± 0.013 ^c^

**270 kg N/ha**	Leaf	0.19 ± 0.04 ^d^	0.017 ± 0.027 ^d^
	Stem	0.16 ± 0.04 ^d^	0.015 ± 0.012 ^d^
	Root	0.11 ± 0.02 ^d^	0.013 ± 0.007 ^d^

All analyses are mean ± standard error of mean (SEM). *N* = 18. Means not sharing a common letter were significantly different at *P* ≤ 0.05.

**Table 5:** DPPH scavenging activities in different parts of *L. pumila* under different nitrogen levels. BHT and α-tocopherol were used as positive controls.

Nitrogen levels	Extract source	Inhibition % a
**0 kg N/ha**	Leaves	61.3 ± 1.6 ^c^
	Stems	57.1 ± 1.1 ^c^
	Roots	51.2 ± 1.0 ^c^

**90 kg N/ha**	Leaves	50.8 ± 1.0 ^d^
	Stems	48.1 ± 0.9 ^d^
	Roots	46.7 ± 0.4 ^d^

**180 kg N/ha**	Leaves	46.4 ± 0.2 ^e^
	Stems	42.7 ± 0.9 ^e^
	Roots	40.2 ± 1.2 ^e^

**270 kg N/ha**	Leaves	37.2 ± 2.2 ^f^
	Stems	32.1 ± 1.2 ^f^
	Roots	30.6 ± 3.2 ^f^

**Controls**	BHT	65.6 ± 1.3 ^b^
	α-tocopherol	76.3 ± 1.2 a

All analyses are mean ± standard error of mean (SEM); *N* = 18. Means not sharing a common single letter are significantly different at *P* ≤ 0.05.

a Results expressed in percent of free radical inhibition.

**Table 6:** Total antioxidant (FRAP) activity in different parts of *L. pumila* under different nitrogen levels. BHT, α-tocopherol and vitamin C were used as positive controls.

Nitrogen levels	Extract source	FRAP a
**0 kg N/ha**	Leaves	890.3 ± 11.2 ^c^
	Stems	870.1 ± 13.5 ^c^
	Roots	810.2 ± 21.3 ^c^

**90 kg N/ha**	Leaves	768.0 ± 27.9 ^d^
	Stems	713.8 ± 34.5 ^d^
	Roots	701.4 ± 78.1 ^d^

**180 kg N/ha**	Leaves	617.3 ± 24.7 ^e^
	Stems	589.2 ± 11.3 ^e^
	Roots	534.1 ± 23.3 ^e^

**270 kg N/ha**	Leaves	435.2 ± 24.1 ^f^
	Stems	412.3 ± 11.2 ^f^
	Roots	399.4 ± 24.5 ^f^

**Controls**	BHT	81.3 ± 56.3 ^g^
	α-tocopherol	953.0 ± 45.6 ^b^
	Vitamin C	3301.2 ± 34.6 a

All analyses are mean ± standard error of mean (SEM); *N* = 18. Means not sharing a common single letter are significantly different at *P* ≤ 0.05.

a Results expressed in percent of free radical inhibition.

**Table 7:** Nitrogen fertilization levels of *Labisia pumila* Benth. during the experiment.

Nitrogen (kg N/Ha) 1,2	Total nitrogen fertilizer per plant 3 (g)
0	0.00
90	0.36
180	0.72
270	1.08

1 Nitrogen source used was urea (46% N);

2 Every nitrogen treatment received TSP (Triple super phosphate; 46% P) and MOP (muriate of potash; 60% K) at a standard rate of 180 kg N ha^−1^; the nitrogen was split into three fertilization phases, and each phase was about 33.3% of total nitrogen fertilizer;

3 Every nitrogen treatment receives TSP (triple super phosphate; 46% P; 0.72 g per plant) and MOP (60% K; 0.51 g per plant) at standard rates of 180 kg N/ha.
